# Hormonal Imbalance as a Prognostic Factor of Physical Development of Children with Intellectual Disability

**DOI:** 10.3390/children11080913

**Published:** 2024-07-29

**Authors:** Olga V. Smirnova, Elizaveta S. Ovcharenko, Edward V. Kasparov

**Affiliations:** Scientific Research Institute of Medical Problems of the North, Separate Division of Federal Research Centre “Krasnoyarsk Science Centre” of the Siberian Branch of Russian Academy of Science, 660022 Krasnoyarsk, Russia; liza-bet@bk.ru (E.S.O.); inpm@inpm.ru (E.V.K.)

**Keywords:** intellectual disability, autonomic regulation, hormones, physical development

## Abstract

Introduction: The purpose was to study the indicators of physical development of primary-school-aged children with intellectual disability by observing the type of autonomic nervous regulation and their levels of catecholamines and serotonin. Methods: A total of 168 primary school age children were examined, of which 54 had intellectual disability. The autonomic nervous system was assessed using cardiointervalography; anthropometric parameters were applied in accordance with recommendations. The contents of serotonin and catecholamines in blood plasma and lymphocytes were assessed using enzyme immunoassay and luminescent histochemical methods. Results and conclusions: Delayed physical and mental development in children with intellectual disability were associated with low serotonin levels in this group of children. The optimal option for the physical development of children with intellectual disability is a sympathetic type of autonomic nervous regulation, while negative-type vagotonic nervous regulation was associated with the maximum delay in physical development. The hypersympathetic type of nervous regulation was accompanied by minimal changes in physical development, despite the hormonal imbalance in the ratio of catecholamines and serotonin. The level of the neurotransmitter serotonin is a prognostic marker of the physical development of children of primary school age. The total amount of catecholamines and serotonin in blood plasma has a direct relationship with the amount of these neurotransmitters in blood lymphocytes; the more hormones in plasma, the more of them in lymphocytes. Therefore, the determination of the contents of catecholamines and serotonin in lymphocytes can be used as a model for studying neurotransmitters in humans.

## 1. Introduction

One of the indicators of children’s health is physical development. The correspondence of anthropometric indicators to the proper values indicates the harmony of the child’s physical development, the correctness of its formation in a certain period of childhood. Physical development represents a dynamic process of growth (increase in body height and weight, and development of organs and body systems) and biological maturation of a child [1]. Primary school age is regarded as one of the most sensitive periods of ontogenesis. Children of this age group are characterized by immaturity of morphofunctional characteristics and increased vulnerability to the effects of adverse environmental factors. Moreover, this particular age range coincides with the beginning of systematic education, characterized by a change in daily routines, a reduction in physical activity, and significant emotional and intellectual stress on the child’s body [2], which often negatively affects the health of children. The concept of intellectual disability suggests that an individual has limitations in their ability to learn at an expected level and in everyday life, and in the case of children, this may cause them to develop at a slower rate than the rest of their peers of the same age. Violation of a child’s cognitive, behavioral and emotional functions due to brain damage is accompanied by a violation of their physical development. Intellectual disability is diagnosed in children under 18 years of age and is characterized by the presence of a number of limitations in adaptive behavior, both at the level of practical, social, and conceptual skills necessary for functioning in everyday life with impaired intellectual functioning [3,4]. Physical and mental development are interconnected. A number of studies have shown that the better the physical condition of the body and the higher the level of physical activity, the better the cognitive abilities of children and adolescents [5,6]. At the same time, physical development is certainly more labile in childhood, and so changes and anomalies appear immediately, whereas mental development is a long and often hidden process, significantly dependent on the age characteristics of the individual. The hormonal background of the child is important for the balanced physical and mental development of the child. Many works have been devoted to the study of the influence of somatotropic hormone, thyroid hormones, etc., on the growth and development of children. Studies of serotonin in terms of its role in the formation of the physical component are mainly carried out for various deviations in physical development (for example, obesity) [7]. At the same time, serotonin is considered a regulator of psychological characteristics, without taking into account its involvement in the activity of the parasympathetic division of the autonomic nervous system. Physical and mental development require a developed autonomic nervous system, but also an adequate level of the hormones that characterize it. Optimal for the growth and vital activity of children of primary school age is the eutonic type of nervous regulation, with a balanced content of catecholamine hormones and hormones of vagotonic action. Recently, serotonin has been considered a hormone of vagotonic action, but there is not enough work assessing its effect on the physical development of children of primary school age. The purpose was to study the indicators of physical development of children of primary school age with intellectual disability, depending on the type of autonomic nervous regulation and the contents of the hormones catecholamines and serotonin. We hypothesize that autonomic nervous regulation has a direct impact on physical development. We assume that the delay in the physical development of children of primary school age with intellectual disability is due not only to other variants of autonomic nervous regulation, but also to a lack or imbalance of hormones of autonomic nervous regulation itself.

## 2. Methods

### 2.1. Study Design

We conducted a cross-sectional (single-stage) case–control study from May 2017 to June 2018. This study was approved by the local bioethical committee of the Research Institute of Medical Problems of the North of the Federal Research Center KSC SB RAS (protocol No. 4 of 10 April 2017). In the examined children, ethical principles required by Art. 24 of the Constitution of the Russian Federation and the Declaration of Helsinki by the World Medical Association were followed (1964, last revision 2013, Fortaleza, Brazil).

### 2.2. Participants

Initially, 209 children of primary school age (7–11 years old) were included in the study. Of these, 95 were children with intellectual disability, and 114 were intellectually healthy children. However, 41 children with intellectual disability were excluded from the study due to exclusion criteria. The final sample consisted of 168 children, of which 54 were children (21 girls and 33 boys, average age 9.1 ± 1.2 years) with intellectual disability (main group) studying in specialized correctional schools No. 3, No. 4, and No. 5 of Krasnoyarsk city. The criteria for inclusion in the main group were as follows: diagnosis of “intellectual disability” (F70, F71), obtaining informed consent to participate in the study from the child’s official representatives. The diagnosis of intellectual disability was made by a qualified psychiatrist during a periodic medical examination of pupils in accordance with the International Classification of Diseases, 11th revision (ICD-11). The exclusion criteria from the main group included the following: receipt of specific immunotherapy 2 months before the start of the examination, the presence of acute or exacerbation of chronic diseases at the time of the examination, residence in the area for less than three years, a history of diagnosis of “behavioral disorder”, “hydrocephalic syndrome”, “convulsive syndrome”, or “cerebral palsy”, the presence of diseases that disrupt the central nervous system (for example, bronchial asthma, epilepsy, migraine, or diabetes), and refusal of the child’s official representative to participate in the study.

The control group included 114 children (46 girls and 68 boys, average age 9.1 ± 1.2 years) studying at secondary school No. 14 in Krasnoyarsk city. The criteria for inclusion in the comparison group were as follows: children of I-II health groups, primary school age (7–11 years), and obtained informed consent to participate in the study from the child’s official representatives. The exclusion criteria from the comparison group included the following: receipt of specific immunotherapy 2 months before the start of the examination, presence of acute or exacerbation of chronic diseases at the time of examination, residence in the area for less than three years, and refusal of the child’s official representative to participate in the study.

**Figure 1:**
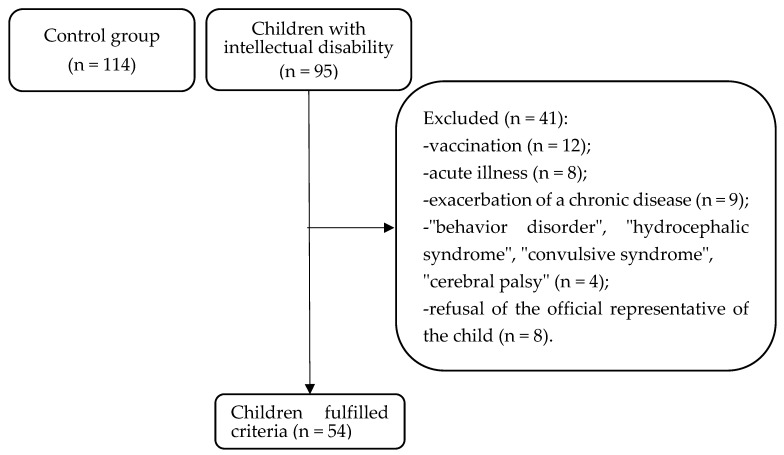
Study design.

**Figure 2:**
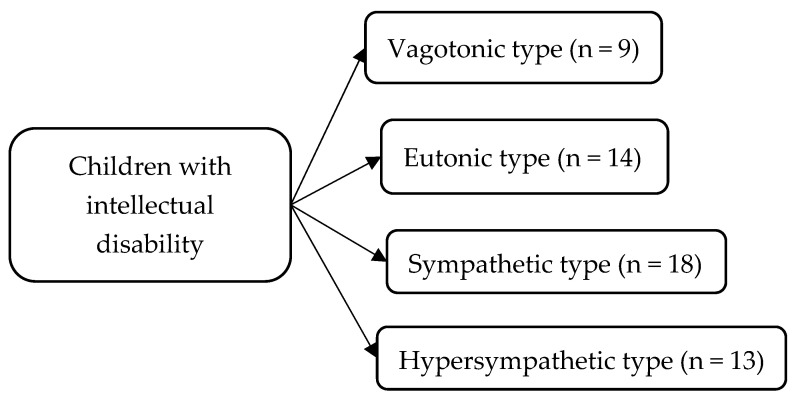
Characteristics of children with intellectual disability depending on the predominant type of autonomic nervous regulation.

**Figure 3:**
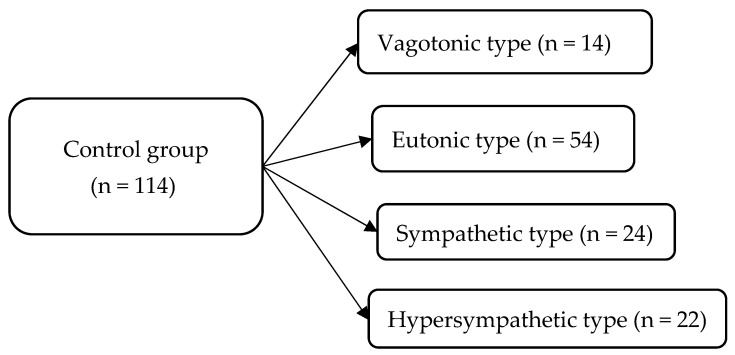
Characteristics of children in the control group depending on the predominant type of autonomic nervous regulation.

In accordance with the goal, the study design (Figure 1) and the distribution of children depending on the predominant type of autonomic nervous regulation in the group of children with intellectual disability (Figure 2) and in the control group (Figure 3) are presented below.

### 2.3. Assessment of Initial Autonomic Tone

The state of the vegetative nervous system (VNS) was assessed by the method of cardiointervalography using the ORTO Valeo software 2.5 and hardware complex (NPP “Living Systems”, Kemerovo, Russia). Cardiointervalography is based on the registration of sinus heart rhythm and mathematical analysis of its structure. An active orthostatic test was used as a stress test, which involved changing the body position from horizontal (lying on the back) to vertical. In this case, a number of physiological changes in the cardiovascular system were observed: the redistribution of blood under the influence of gravity (from the chest to the abdominal cavity and lower extremities), which was accompanied by a decrease in cardiac output, central and minute blood volume, and blood pressure. It is the decrease in blood pressure that promotes irritation of the mechanoreceptors of the baroreflexogenic zones, triggering compensatory reactions in the form of an increase in heart rate and increased vascular tone, due to the activation of the sympathetic division of the autonomic nervous system [8]. Heart-rate recording was carried out in the first half of the day, in the absence of significant physical and intellectual stress before the experiment. The subject took a horizontal position (lying on their back), and after a 10-min rest, cardiac cycles were recorded (at least 100 consecutive R–R intervals). After this, the subject calmly stood up (without leaning on the couch), and another 210 cardio intervals were recorded.

Based on the cardiointervalography data, the stress index (SI, conventional units) was calculated:

SI = (AMo (%))/(2 × Mo × ΔX),

where Mo (mode, s) is the most frequently occurring value of cardiointervals in the dynamic series, AMo (mode amplitude, %) is the number of interval values corresponding to the mode and expressed as a percentage of the total number of cardiocycles of the array, and ΔX (variation range, s) is the difference between the maximum and minimum values of the duration of the RR intervals in a given array of cardiac cycles.

To assess the background state of autonomic regulation, we used the determination of initial autonomic tone (IAT) at rest [9]. IAT was assessed using the stress index (SI) [10]. Depending on the value of SI, the following types of initial autonomic tone were distinguished:

SI < 30 conventional units—vagotonic-type (VT) IAT;

SI from 31 to 90 conventional units—eutonic-type (ET) IAT;

SI from 91 to 160 conventional units—sympathetic-type (ST) IAT;

SI > 161 conventional units—hypersympathetic type (HT) of IAT.

The initial autonomic tone at rest was regarded as the dominant variant of nervous regulation in the child.

### 2.4. Assessment of Hormonal Activity

The contents of serotonin and catecholamines were determined in blood plasma (total amount) and in lymphocytes (as a result of their reuptake). The contents of serotonin and catecholamines in blood plasma were determined by enzyme immunoassay on an ELISA analyzer, Thermo Scientific Multiskan FC (Thermo Fisher Scientific, Walten, MA, USA), using research kits (Vector Best, Novosibirsk, Russia). The contents of serotonin and catecholamines in blood lymphocytes were assessed using the luminescent histochemical method according to Yokoo et al. (1982) as modified by Novitskaya (2000) [11]. This method is based on the reaction of biogenic monoamines with formaldehyde vapor to form a luminescent complex that produces green fluorescence.

The preparatory stage included the following steps: (a) blood smears were prepared with a 5% solution of magnesium sulfate (MgSO4) (in a 1:1 ratio) on a non-fluorescent glass slide; (b) the smears were dried in a hot air stream (for 15 min); (c) the smears were placed in a vacuum desiccator (1 atm) for 30 min; (d) the required amount of paraformaldehyde (PJSC “Uralchimplast”, Ekaterinburg, Russia) was weighed (for serotonin, 1.450 g; for catecholamines, 1.244 g), which was added to a desiccator (volume 1.7 L) with distilled water (for serotonin, 441 µL; for catecholamines, 350 µL) and heated at a temperature of 80 °C for 15 min; and (e) blood smears from a vacuum desiccator were transferred to a desiccator with paraform and incubated at a temperature of 80 °C for 1 h (for catecholamines) or for 2 h (for serotonin).

The processed smears were examined using a fluorescent microscope “LYUMAM-I3” (LOMO, Saint Petersburg, Russia) with an attachment, “FMEL-1” (voltage 700 V, resistance 5 × 10^−6^ Ohm). The signal was outputted to a digital voltmeter, and the reference background was measured on a standard target glass. In each smear, the fluorescence of 15 cells was measured, the average value was calculated, and the average value of the background fluorescence taken at three points of the smear was subtracted. The fluorescence signal in μV obtained on a voltmeter was expressed in conventional units.

In our study, we did not isolate individual types of catecholamines, but studied them together. There are three main catecholamines present in plasma—norepinephrine, dopamine, and adrenaline. The main neurotransmitter of the sympathetic nervous system is norepinephrine, and its regulatory action is inextricably linked with adrenaline. It is believed that norepinephrine and adrenaline, despite some differences in their functions, are combined into a single sympatho-adrenal system, which ensures the functioning of the body under stressful conditions [12]. The activation of the sympathetic nervous system leads to the release of norepinephrine from sympathetic fibers, which in turn promotes the release of adrenaline from the adrenal glands [13]. Dopamine is predominantly a central neurotransmitter, and its plasma concentration is negligible [14]. The action of each neurotransmitter complements the action of the other and collectively shows the influence of sympathetic nervous regulation on all human systems and organs. In accordance with the above and the task set, we consider it advisable to study all catecholamines simultaneously.

### 2.5. Assessment of Physical Development Parameters

The assessment of anthropometric parameters was carried out in accordance with the methodological recommendations of the Russian Association of Endocrinologists (2017) [1]. Anthropometric measurements were carried out in the morning in the school’s medical offices at a comfortable temperature, using standardized instruments in accordance with the used methods. Body height was measured with a vertical stadiometer RM-2 (Diakoms LLC, Moscow, Russia) with a division value of 0.1 cm. Body weight was determined using mechanical medical scales SECA 700 (Hamburg, Germany) with an accuracy of 0.1 kg. Based on anthropometric parameters, body mass index was calculated as the ratio of body weight (kg) to body height (in meters) squared (m^2^). The circumference of the chest and head was assessed in a standing position using a rubberized measuring tape (the tape was changed every 100 measurements). To measure the circumference of the chest, a centimeter tape was placed at the back at the angles of the shoulder blades, and in front at the level of the nipples. When measuring head circumference, a centimeter tape was used to tightly grasp the occipital protuberance from behind and the brow ridges from the front. To measure the transverse diameter of the chest, the legs of a large thick Kafa compass (Moscow, Russia) were placed on the ribs in the axillary region at the level of the midsternal point (the fourth rib), at the site of the greatest protrusion of the lateral parts of the ribs.

### 2.6. Statistical Analysis

Statistical data processing was carried out using the Statistica 10 application package (StatSoft Inc., Tulsa, OK, USA). The normality of the distribution was checked using the Shapiro–Wilk test, followed by an assessment of the equality of variances using the Levene test. Since the data did not obey a normal distribution, statistical differences between samples were identified for quantitative characteristics using nonparametric statistic methods—the Kruskal–Wallis test, the Mann–Whitney U test, and the calculation of the median (Me) and interquartile range (C_25_–C_75_). To determine the significance of differences between one characteristic and another, the frequency method (χ-square) was used. The strength of association between variables was calculated using Spearman’s correlation test. The critical level of significance when testing statistical hypotheses was *p* < 0.05 [15].

## 3. Results

Previously, we found that in children with intellectual disability, the sympathetic regulation of physiological functions dominates: 33% of children of primary school age with intellectual disability have a sympathetic type of initial autonomic tone, 24% have a hypersympathetic type of IAT, while the optimal type of regulation (eutonic) is found only in 26% of children in this group [16].

In this study, in the first stage, the contents of serotonin and catecholamines, as mediators of two parts of the autonomic nervous system, were assessed (Table 1). It was found that in the control group and the group of children with intellectual disability, the content of catecholamines in plasma and lymphocytes was proportional to the predominant type of autonomic regulation—with the sympathetic and hypersympathetic types of IAT, the content of catecholamines is statistically significantly higher than that of the vagotonic type (*p* < 0.05). This corresponds to the importance of catecholamines as the main mediators of the sympathetic part of the autonomic nervous system [17,18].

With regard to serotonin, in both groups, a decrease in the content of this indicator was recorded in plasma and lymphocytes as the influence of the sympathetic component of the ANS increased: in the control group, a statistically significant decrease in serotonin was detected with the hypersympathetic type of IAT in the group with intellectual disability, with the sympathetic type of IAT relative to the vagotonic type of the initial autonomic tone (Table 1). Recently, more and more data have appeared on the participation of serotonin in the regulatory activity of the autonomic nervous system [18,19,20]; this may explain the features we identified. It is worth noting that the serotonin content was reduced in plasma and lymphocytes in children with intellectual disability compared to the control group regardless of the type of autonomic nervous regulation. The content of catecholamines in the sympathetic and hypersympathetic types of IAT in children with intellectual disability was significantly higher in plasma and lymphocytes compared to the control group (*p* < 0.05) (Table 1).

The catecholamine/serotonin ratio in both groups also reflects the balance of activity of the sympathetic and parasympathetic parts of the autonomic nervous system—we recorded an increase in this ratio as sympathetic activity increased. Moreover, this coefficient was statistically significantly higher in the group of children with intellectual disability with eutonic, sympathetic, hypersympathetic types of initial autonomic tone relative to the control group (Table 1).

Thus, in children with intellectual disability with vagotonic and eutonic types of nervous regulation, a violation of hormonal regulation was recorded in the form of a decrease in serotonin levels in plasma and lymphocytes, whereas in the sympathetic and hypersympathetic types of autonomic nervous regulation, a hormonal imbalance was revealed, characterized by a reduced level of serotonin, as well as increased levels of catecholamines and the catecholamine/serotonin ratio compared to the control group. It has been proven that serotonin is necessary for prenatal and postnatal brain development [21,22]; various deviations in the serotonergic system are associated with the formation of developmental disorders of the mental, cognitive, emotional sphere (autism, intellectual disability, syndrome attention deficit, etc.) [23,24,25]. The revealed low serotonin content in plasma and lymphocytes in children with intellectual disability, regardless of the type of nervous regulation, corresponds to the characteristics of the development of the nervous system in children of this group.

In the second stage, a study of anthropometric indicators in the studied children was carried out depending on the predominant type of autonomic nervous regulation. In the control group, the body-height indicator ranged from 135 with a hypersympathetic type of autonomic regulation (minimum value) to 138.3 cm with a vagotonic type of autonomic regulation (maximum value), although no statistically significant differences were found. The minimum body weight in the control group was recorded for the vagotonic and the sympathetic (30 kg) type of IAT; the maximum value was noted for the hypersympathetic type of IAT (32.6 kg). According to the body mass index, a trend opposite to body height was revealed—the minimum value was established for the vagotonic type (15.1 kg/m^2^) and the maximum for the sympathetic type of IAT (16.9 kg/m^2^), although no statistical differences were established. Indicators of the transverse diameter of the chest and chest circumference also tended to increase from the vagotonic to the sympathetic type of autonomic regulation. Thus, it can be noted that indicators of physical development in healthy children of primary school age generally developed (formed) autonomously (independently) from the functioning of the autonomic nervous system (Table 2).

In the group of children with intellectual disability, the minimum body-height indicators were recorded with a vagotonic type of initial autonomic tone. In the eutonic and sympathetic types of IAT, body-height indicators were statistically significantly higher relative to the vagotonic type. For all other anthropometric indicators, the same dynamics of growth from the vagotonic type (minimum values) to the sympathetic type of IAT (maximum statistically significant values) were recorded. With the hypersympathetic type of nervous regulation, the values of body weight, BMI, head circumference, and transverse diameter of the chest were statistically significantly higher than those of the vagotonic type (Table 2).

When comparing children with intellectual disability and the control group, depending on the type of nervous regulation, it was found that if with the vagotonic and eutonic types of regulation, anthropometric parameters in the group of children with intellectual disability were statistically lower than the control group, then with the sympathetic type of nervous regulation, the anthropometric parameters in children with intellectual disability significantly exceeded the indicators of the control group (*p* < 0.05) (Table 2). In general, it is worth noting that in children with intellectual disability, physical development depends on the characteristics of autonomic nervous regulation. Despite some autonomy, the autonomic nervous system is under the control of a number of central parts (hypothalamus, prefrontal cortex, and limbic system) [26]. It can be assumed that the disruption of the functioning of the central nervous system in children with intellectual disability is reflected in the processes of autonomic regulation, which in turn manifest themselves in the disruption of the formation of the child’s physical status.

Thus, with vagotonic and eutonic types of autonomic nervous regulation, unidirectional changes in the parameters of physical development were recorded—in children with intellectual disability, the parameters of body height, body weight, body mass index and head circumference were reduced compared to the control group. In this case, the maximum number of changes was recorded with the vagotonic type of nervous regulation. The obtained data on anthropometric parameters coincide with the low level of serotonin detected in vagotonic and eutonic types of nervous regulation in children with intellectual disability. Serotonin is known to play an important role in the growth and development of the body [27]. With the predominant sympathetic type of regulation, on the contrary, indicators of body weight, body mass index and chest circumference in children with intellectual disability exceed those of children in the control group, which may be due to the influence of the sympathetic part of the autonomic nervous system on metabolism and the formation of body composition through the production of catecholamines [28,29]. At the same time, with the hypersympathetic type of autonomic nervous regulation in children with intellectual disability, despite the imbalance in the contents of catecholamines and serotonin, anthropometric indicators correspond to the proper values of children in the control group.

**Figure 4:**
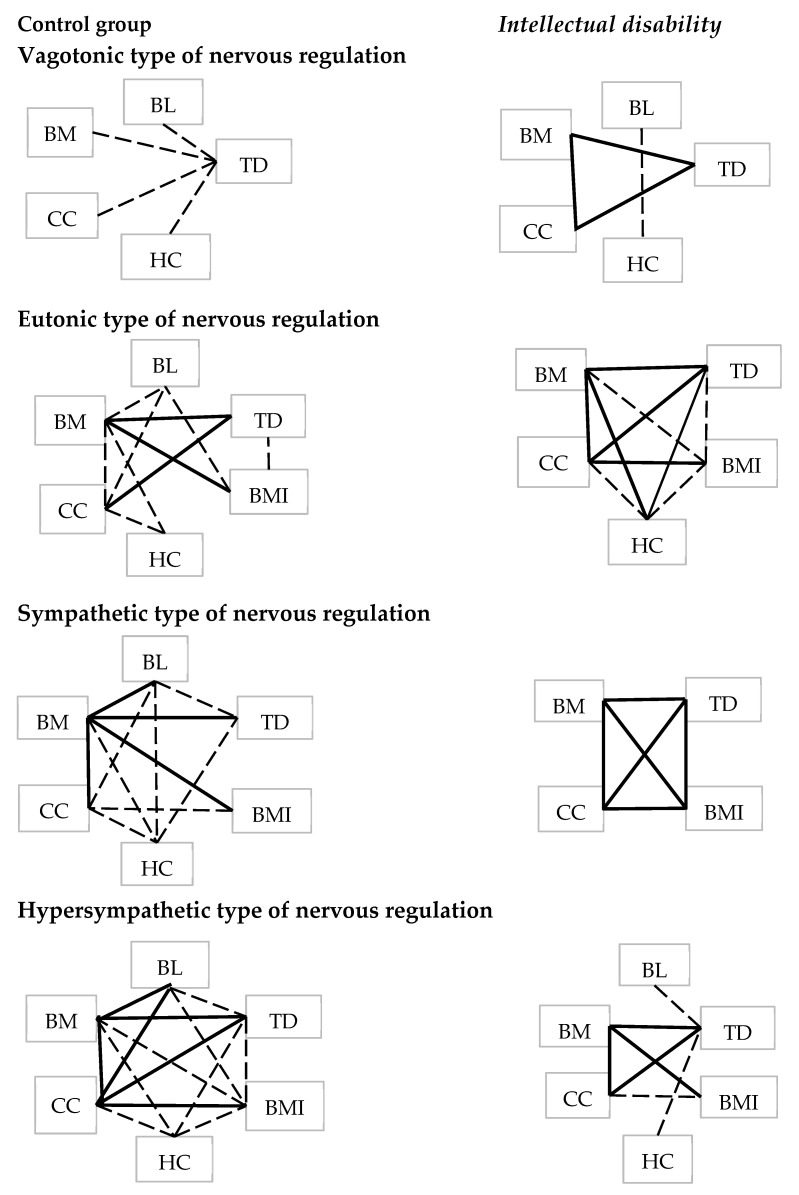
Intrasystem correlation relationships of anthropometric parameters in the studied groups. Interrelations of moderate and strong force are presented (*p* < 0.05). BL—body height, BM—body mass, CC—chest circumference, HC—head circumference, BMI—body mass index, TDC—transverse diameter of the chest, —Strong correlation (0.7–1.0), ——medium-strength correlation (0.3–0.69).

When analyzing the intrasystem relationships of physical development parameters in the control group, as expected, direct correlations of anthropometric indicators were revealed (*p* < 0.05) (Figure 4). With the optimal type of nervous regulation (eutonic), 10 correlation relationships were recorded, with body weight having the largest number of connections with other anthropometric parameters. As the influence of the sympathetic component of the ANS increased, the number of connections increased (with the sympathetic type of nervous regulation—11 connections, with the hypersympathetic type—14). An increase in the relationships between anthropometric parameters with an increase in the dominant influence of the sympathetic department of the autonomic nervous system indicates an increase in tension in the body, which is physiologically justified, since it is the sympathetic part of the ANS that ensures the mobilization of all the body’s resources under the influence of internal or external stress factors. With the vagotonic type of nervous regulation, a minimum number of correlations were recorded (Figure 4).

In the group of children with intellectual disability, physical development parameters also had direct relationships with each other (*p* < 0.05) (Figure 4). With the eutonic type of nervous regulation in the group with intellectual disability, the number of correlation relationships of anthropometric parameters is comparable to the control group (10 connections). However, with increasing sympathetic influence, the number of correlation connections is significantly reduced (with the sympathetic type of nervous regulation, six connections were identified; with the hypersympathetic type—seven), which may indicate insufficient coordination of the body systems and the impossibility of an adequate response under stressful conditions.

## 4. Discussion

When conducting this study, we selected parameters used by the autonomic nervous system as peripheral neurotransmitters. For the sympathetic division of the autonomic nervous system, catecholamines exhibit such activity [17,18]. Serotonin was chosen for the parasympathetic component of the ANS, since there is currently evidence of a close relationship between the components of the serotonergic system and the parasympathetic activity of the autonomic nervous system. According to Hildreth et al. (2008) [19], serotonin determines the vagal modulation of heart rate. Low activity of the parasympathetic part of the autonomic nervous system against a background of low serotonin levels has been recorded in a number of studies [30,31,32]. According to Lin et al. (2019) [33], tryptophan deficiency decreases the high-frequency component (HF) of heart rate and increases the low-frequency component (LF) of heart rate. It is also important that catecholamines and serotonin perform key regulatory functions of metabolic processes in the body [34,35].

Indicators of healthy children were used as normative indicators of the contents of catecholamines and serotonin for different types of autonomic nervous regulation. In general, it can be noted that the content of catecholamines is proportional to the level of activity of the sympathetic department in a given group. The serotonin content in healthy children decreased as the influence of the parasympathetic division of the ANS decreased, which confirms previous studies [19,30,31,32] and makes it possible to use serotonin as a mediator of the parasympathetic division of the autonomic nervous system.

In the group of children with intellectual disability, serotonin content also decreased in proportion to the level of parasympathetic activity. However, when compared with the control group, a sharp lag in this indicator was recorded, regardless of the type of initial autonomic tone. Serotonin has been found to play a critical role in the development of the brain and nervous system as a whole, and its levels are reduced in a number of neuropsychiatric diseases [36,37]. Also, in the group of children with intellectual disability with sympathetic and hypersympathetic types of nervous regulation, there was a significant predominance of catecholamines compared to the control group. In general, in children with intellectual disability, the contents of catecholamines and serotonin are proportional to the activity of the corresponding part of the autonomic nervous system, but have a sharp imbalance relative to the proper values in the control group.

Analysis of the parameters of physical development in children with intellectual disability with vagotonic and eutonic types of nervous regulation revealed a lag in anthropometric indicators from the parameters of the control group, which, in our opinion, may be due to the low level of serotonin identified in these groups. Considering that serotonin is an important factor for optimal brain development, and head circumference is recognized as an indicator not only of brain development but also of the level of intellectual ability [38,39,40], the lag in head circumference we identified in children with intellectual disability proves the presence of brain damage in children of this group. When analyzing correlation interactions of physical development parameters in children with intellectual disability, head circumference is somewhat independent from other anthropometric indicators. In addition, the influence of serotonin on the growth and development of a child, starting from the prenatal period, has been proven. It has been established that a lack of placental serotonin is accompanied by delayed growth and development of the fetus [25]. It has also been noted that serotonin and its metabolites (in particular tryptophan) stimulate the production of growth hormones [41,42,43], which is necessary for normal growth and maturation body, which may explain the reduced body height and weight we identified in children with intellectual disability.

In the group of children with intellectual disability, the sympathetic type of nervous regulation had a positive effect on indicators of physical development—a predominance of all anthropometric parameters was found, with the exception of head circumference, compared to the control group. The sympathetic division of the autonomic nervous system is primarily known for its role in mediating the fight-or-flight response and is essential for body homeostasis. Sympathetic axons innervate peripheral organs and tissues throughout the body to control various physiological processes, including cardiac output, body temperature, blood glucose level, and immune function under normal conditions and in response to external stressors [44]. The sympathetic regulation of organ function is based on close contacts between neurons and targets established during embryonic and postnatal development [45]. Sympathetic dysfunction is associated with a number of human diseases, including peripheral neuropathies, heart failure, hypertension, and diabetes, some of which may be genetic in origin [46,47]. There is also emerging evidence that sympathetic innervation regulates stem-cell niches to promote tissue regeneration through the deployment of developmental signaling pathways [48]. Sympathetic neurons are remarkably heterogeneous and are characterized by the acquisition of molecular and cellular diversity necessary to meet the functional needs of various peripheral organs and their differentiation occurring during embryonic and postnatal development [49]. The establishment of functional circuits of sympathetic neurons involves coordinated interactions between neurons, glial cells, and peripheral tissues. The aberrant development of sympathetic neurons may contribute to the etiology of a number of diseases, including psychiatric disorders, familial dysautonomia [50,51], Down syndrome [52], cardiac and metabolic dysfunctions [46,53,54,55]. This explains the optimality of the sympathetic type of autonomic nervous regulation for the physical development of children with intellectual disability.

An interesting fact is that with hypersympathetic types of autonomic nervous regulation, the anthropometric indicators of children with intellectual disability corresponded to the average indicators of physical development of children in this group. Probably, under conditions of excessive sympathetic influence, tolerance to the neurotransmitters catecholamines develops, accompanied by a slowdown in physical development.

Thus, serotonin deficiency is critical in the formation of delayed intellectual and physical development, which is manifested by low anthropometric indicators in children with intellectual disability with vagotonic and eutonic types of autonomic regulation and is confirmed by the role of central and peripheral serotonin in the development of the body. However, with a simultaneous high content of catecholamines, disharmony of physical development is observed with an increase in indicators of physical development in comparison with the proper values of healthy children. In our opinion, identifying the relationship between the parameters of autonomic regulation, neurotransmitter systems and physical development in the teenage age group in the context of hormonal changes occurring during this period may be promising.

Limitations of the study included children with hereditary defects, brain injuries, severe somatic diseases, and brain-development anomalies.

## 5. Conclusions

Delayed physical and mental development in children with intellectual disability were associated with low serotonin levels in this group of children.

The optimal option for the physical development of children with intellectual disability was the sympathetic type of autonomic nervous regulation.

The hypersympathetic type of nervous regulation was accompanied by minimal changes in physical development, despite the hormonal imbalance in the ratio of catecholamines and serotonin.

A decrease in head circumference was detected in children with intellectual disability with vagotonic and eutonic types of autonomic nervous regulation.

An extremely negative type of autonomic nervous regulation—vagotonic—was associated with the maximum delay in physical development in children with intellectual disability.

The amount of the neurotransmitter serotonin is a prognostic marker of the physical development of children of primary school age.

The total contents of catecholamines and serotonin in blood plasma have a direct relationship with the amounts of these neurotransmitters in blood lymphocytes; the more hormones in plasma, the more of them in lymphocytes. Therefore, the determination of the contents of catecholamines and serotonin in lymphocytes can be used as a model for studying neurotransmitters in humans.

## Figures and Tables

**Table 1:** Content of catecholamines and serotonin in plasma/in lymphocytes depending on the type of autonomic nervous regulation in children with intellectual disability compared to the control group (Me; C_25_–C_75_).

		Control Group		Intellectual Disability		Statistical Significance
		In Plasma (ng/mL)	In Lymphocytes (Conventional Units)	In Plasma (ng/mL)	In Lymphocytes (Conventional Units)	
Vagotonic-type NR	Catecholamines	27.54 [25.84–37.91]	16.2 [15.2–22.3]	29.58 [28.56–40.29]	17.4 [16.8–23.7]	
	Serotonin	123.76 [82.96–142.8]	36.4 [24.4–42]	76.5 [67.32–85.68]	22.5 [19.8–25.2]	0.023
	Catecholamines/Serotonin	0.27 [0.22–0.31]	0.48 [0.4–0.79]	0.42 [0.39–0.47]	0.85 [0.76–1.1]	
Eutonic-type NR	Catecholamines	30.46 [20.16–40.76]	17.4 [15.3–20.7]	32.3 [27.88–37.23]	19 [16.4–21.9]	
	Serotonin	125 [51–199]	34 [27.3–43.9]	77.86 [54.74–97.24]	22.9 [16.1–28.6]	0.00002
	Catecholamines/Serotonin	0.24 [0.2–0.4]	0.5 [0.49–0.67]	0.41 [0.38–0.51]	0.83 [0.65–1.24]	0.00012
Sympathetic-type NR	Catecholamines	30.94 * [28.39–32.81]	18.2 * [16.7–19.3]	38.08 ** [30.43–45.39]	22.4 ** [17.9–26.7]	0.041
	Serotonin	108.8 [100.64–123.76]	32 [29.6–36.4]	63.24 ** [52.02–77.52]	18.6 ** [15.3–22.8]	0.000004
	Catecholamines/Serotonin	0.28 [0.26–0.28]	0.53 [0.49–0.63]	0.6 *** [0.58–0.85]	1.07 *** [0.96–1.28]	0.000006
Hypersympathetic-type NR	Catecholamines	34.68 * [30.26–38.25]	20.4 * [17.8–22.5]	39.95 ** [34.68–44.03]	23.5 ** [20.4–25.9]	0.018
	Serotonin	102 * [94.86–149.6]	30 * [27.9–44]	64.6 [61.2–77.52]	19 [18–22.8]	0.000011
	Catecholamines/Serotonin	0.34 [0.25–0.36]	0.68 [0.42–0.7]	0.71 [0.57–0.79]	0.98 [0.89–1.5]	0.0003

Note: * statistical significance of the differences compared to the vagotonic type of nervous regulation in the control group (*p* < 0.05); ** statistical significance of differences compared to the vagotonic type of nervous regulation in the group with intellectual disability (*p* < 0.05); *** statistical significance of differences compared with the eutonic type of nervous regulation in the group with intellectual disability (*p* < 0.05).

**Table 2:** Parameters of physical development depending on the type of autonomic nervous regulation in children with intellectual disability compared to the control group (Me; C_25_–C_75_).

Parameter	Vagotonic-Type NR		Eutonic-Type NR		Sympathetic-Type NR		Hypersympathetic-Type NR	
	Control Group (n = 14)	Intellectual Disability (n = 9)	Control Group (n = 54)	Intellectual Disability (n = 14)	Control Group (n = 24)	Intellectual Disability (n = 18)	Control Group (n = 22)	Intellectual Disability (n = 13)
	1	2	3	4	5	6	7	8
Body height, cm	138.3 [129.5–140]	123 [121–130] p1–2 = 0.024	137.3 [132.8–141.5]	127.5 [118–131.5] p3–4 = 0.039 p2–4 = 0.042	135.5 [129–138]	140 [133–145] p2–6 = 0.027	135 [130.5–143]	134.5 [123–146]
Body mass, kg	30 [27–33.7]	23 [22–26] p1–2 = 0.032	31.5 [27.8–34]	29.5 [25–45] p2–4 = 0.032	30 [27–33]	39 [29.9–42] p5–6 = 0.039 p2–6 = 0.006	32.6 [26–36.5]	32 [25–34.6] p2–8 = 0.027
Body mass index, kg/m^2^	15.1 [14.7–17.1]	13.8 [12.8–15.7] p1–2 = 0.03	16.4 [15.6–17.8]	15.3 [13.5–16.5] p3–4 = 0.04 p2–4 = 0.044	16.9 [14.2–17.9]	18.8 [16.1–23.9] p5–6 = 0.03 p2–6 = 0.038	16.7 [15.3–19.3]	17.2 [15.8–18.8] p2–8 = 0.041
Head circumference, cm	52.8 [52–53]	50 [49.5–51] p1–2 = 0.002	52.5 [51.6–53.4]	50.8 [49.5–51.8] p3–4 = 0.042	52.6 [51.5–53.3]	52 [50–53.5]	52.6 [52–54.2]	52.3 [51–54] p2–8 = 0.037
Transverse diameter of the chest, cm	19.8 [19–21]	19 [18.2–19.5]	20.1 [19.2–21.1]	20.4 [19.6–22]	20.2 [19.3–20.9]	21.8 [20–22.8] p2–6 = 0.019	20.4 [19–21.2]	20.2 [19.4–21] p2–8 = 0.038
Chest circumference, cm	62.9 [60.8–68]	58.5 [58–64]	64.8 [61.2–69]	63.5 [62–70]	65 [60.5–67.8]	69 [64–75] p5–6 = 0.041 p2–6 = 0.012	64.3 [60.6–70.5]	65.8 [61–68]

## Data Availability

The participants of this study did not give written consent for their data to be shared publicly, so due to the sensitive nature of the research, supporting data are not available.
